# Ethics, Empathy, and Attitudes: Exploring the Mediating Role of Empathy in Social Workers’ Perspectives Toward Disability

**DOI:** 10.3390/bs16091593

**Published:** 2026-09-07

**Authors:** Ertuğrul Hatipoğlu, Hüsamettin Çetin, Kübra Arslan Aldaş

**Affiliations:** 1 Department of Social Work, Faculty of Health Sciences, Hitit University, 19030 Corum, Turkey; 2 Department of Social Work, Faculty of Health Sciences, Kırıkkale University, 71100 Kirikkale, Turkey; hcetin@kku.edu.tr (H.Ç.); kubraarslan@kku.edu.tr (K.A.A.)

**Keywords:** disability, empathy, ethics, social work, attitudes

## Abstract

This study examines the relationships among professional ethical sensitivity, empathy, and attitudes toward individuals with disabilities among active social workers, specifically focusing on the mediating role of empathy as an “emotional bridge.” Employing a cross-sectional correlational survey design, data were collected from 355 social workers. Participants completed validated scales measuring professional ethical sensitivity, empathic tendencies, and attitudes toward disability. A mediation analysis was conducted using Hayes’ PROCESS Macro (Model 4) with 5000 bootstrap samples. The results indicate that professional ethical sensitivity has a significant positive direct effect on both empathy and attitudes toward disability. Furthermore, empathy significantly mediates the relationship between ethical sensitivity and attitudes toward disability. However, the relatively small indirect effect and the low total variance explained suggest that social workers’ professional attitudes are shaped not only by ethical and empathic factors but also by other contextual dynamics not captured in this model. Ultimately, these findings emphasize that theoretical ethics education in the social work curriculum must be integrated with the development of affective capacities, such as empathy, to translate them into tangible, positive professional actions.

## 1. Introduction

Disability is a multidimensional experience that can significantly impact an individual’s physical, emotional, and social well-being, often intersecting with various societal barriers (Girgin-Aykanat & Balcı, 2015). Historically, people with disabilities have experienced profound exclusion and cultural devaluation, leading to systemic discrimination (Demir, 2022; Saetermoe et al., 2001). As O’Hara (2003, p. 166) emphasizes, the stigma associated with disability can sometimes be more destructive than prejudices related to race, belief, or culture. Furthermore, non-disabled individuals frequently view disabilities as an inherent problem rather than recognizing the individual with disabilities themselves (Morris, 1991). Because the personal experiences, beliefs, and attitudes of society play a crucial role in the acceptance and integration of individuals with special needs (Jorm, 2000), addressing these prejudices is a fundamental societal and professional imperative.

The primary mission of social work is to empower individuals, groups, and communities to enhance their social functioning, ensuring that those who are subject to exclusion, discrimination, and oppression can lead fair and equitable lives (Dubois & Miley, 1996; Payne, 2005; Sheafor & Horejsi, 2016; Zastrow, 2016). In this context, as broadly defined in the professional literature (Barker, 1999), the attitudes of social workers are not merely personal dispositions; they are core determinants of practice capability. Positive practitioner attitudes shape competence, accessibility practices, and service priorities, directly influencing the readiness to provide culturally competent care (Garibay, 2019). Conversely, negative, uninformed, or overly risk-averse attitudes erode cultural safety, trust, and equitable care. For instance, studies indicate that social workers’ attitudes toward deafness significantly correlate with their competence in serving Deaf clients, shaping whether practitioners seek training, use interpreters, or adapt communication strategies. Inaccessible or culturally incompetent care driven by poor provider attitudes contributes to lower health literacy and a higher risk of misdiagnosis (Garibay, 2019). Furthermore, when practitioners deprioritize disability-relevant domains such as the sexual well-being of disabled youth, clients experience critical gaps in holistic care, which damages trust, limits their participation in decision-making, and negatively affects their mental and physical health (Goulden, 2025).

To ensure high-quality care and mitigate such negative attitudes, social work relies heavily on its professional ethical values, which guide right actions, common understanding, and practice perspectives (Cılga, 2004; Reamer, 2018; Thompson, 2017). Central to operationalizing these values is the construct of professional ethical sensitivity. Defined as the practitioner’s ability to recognize an ethical dilemma when it arises, comprehend its implications for vulnerable clients, and interpret the situation through the lens of professional values (Nathanson et al., 2011), ethical sensitivity serves as a foundational framework. It functions as an independent variable that directs practitioner decision-making, organizational policy, and social work education (Knevel et al., 2023). In professional settings, ethical decision-making frameworks, such as Guttman’s (2006) six-step method, help practitioners navigate conflicts between professional obligations and personal values. While individual and demographic characteristics often influence how these ethical frameworks are applied in practice (Choudhury et al., 2012; Jones & Watt, 2001; Pierce & Sweeney, 2010), treating professional ethical sensitivity solely as a cognitive checklist or a rigid regulatory framework presents a critical shortcoming. The mere recognition of ethical codes does not guarantee their translation into disability-affirming practice. Gaps frequently arise because cognitive awareness of an ethical issue does not reliably alter implicit attitudes or lived engagement with disabled people (Presnell & Keesler, 2022). Without strong integration into educational pedagogy (Kattari et al., 2020) and institutional enactment that emphasizes disability justice (Presnell & Keesler, 2022), ethical sensitivity often fails to produce measurable changes in service delivery.

This study challenges the reductive perspective of relying solely on the superficial recognition of ethical codes, highlighting that professional ethical sensitivity, when isolated, is insufficient to ensure positive attitudes toward people with disabilities. It is at this juncture that empathy emerges as an indispensable “psychological connective tissue” a mediating mechanism that helps translate abstract ethical principles into genuine professional behavior. Empathy involves the capacity to recognize, understand, and sensitively respond to another person’s emotional state and thoughts (Kohut, 2009; Rogers, 1983). It allows individuals to move beyond self-destructive thoughts and implicit biases (Cooney, 1999) while fostering tolerance, care, and positive mental development (Kalliopuska, 1992). When the emotional resonance provided by empathy is absent, even the most rigorous ethical standards risk becoming hollow or purely theoretical statements. This creates a troubling paradox where practitioners highly versed in professional ethical rules may still unconsciously harbor biases or become complicit in stigmatizing processes. Ultimately, the transition from “knowing what is right” to “feeling what is right and acting upon it” does not occur spontaneously; empathy can serve as an emotional bridge linking professional obligations and concrete practice.

Previous research has examined attitudes toward disability (Cheatham et al., 2015) and explored empathy and ethics separately within social work contexts (Nathanson et al., 2011). While the existing literature establishes that both ethical frameworks and empathetic responses independently shape interpersonal attitudes, a notable empirical gap remains within disability studies. Specifically, it remains under-researched whether professional ethical sensitivity positively influences attitudes toward disability by operating in tandem with empathy. Addressing this gap, this study aims to investigate the structural relationship between social work ethics, empathy, and attitudes toward disability, hypothesizing that empathy serves as a critical mediating variable.

Based on this framework, the following hypotheses were formulated:

**H1.**  *Professional ethical sensitivity is positively correlated with more inclusive attitudes toward disability.*

**H2.**  *Empathy is positively correlated with more inclusive attitudes toward disability.*

**H3.**  *Empathy significantly mediates the relationship between professional ethical sensitivity and attitudes toward disability.*

## 2. Methods

This study employed a cross-sectional relational survey design to investigate whether empathy mediates the relationship between social workers’ ethical orientation and their attitudes toward people with disabilities. The relational survey model is suitable for examining associations among multiple variables and testing mediation hypotheses in a single explanatory framework (Karasar, 2016). While causality cannot be inferred due to the cross-sectional nature, this design allows for identifying statistically significant associations and potential mechanisms linking ethics, empathy, and disability-related attitudes.

### 2.1. Study Group

#### Research Group and Sampling Process

This study was conducted online via Google Forms with social workers who participated on a voluntary basis. The convenience sampling method was adopted for sample selection, and no restrictive exclusion criteria were established for participation. The sample, which represents the study population, consists of a total of 355 professionals, including 240 women (67.6%) and 115 men (32.4%). The average age of the participants was determined to be 32.20; a large majority (61.7%) were single. Regarding the participants’ personal connection to disability, it was found that 1.4% of the participants were personally disabled, 11.8% had a family member with a disability, and 49% were in contact with a person with a disability in their immediate social circle.

In this study, the term “social worker” refers to professionals who have completed a four-year Bachelor of Social Work (BSW) degree and are currently actively working in various social service fields, such as public institutions (e.g., ministries), civil society organizations, or healthcare facilities. Participants were reached through professional networks and digital platforms. Prior to the data collection phase, an “Informed Consent” form outlining the study’s purpose and participants’ right to withdraw at any time was provided digitally, and their consent was obtained. To ensure professional validity, participants’ graduation departments were verified.

All participants were confirmed to be in active practice at the time of data collection. Regarding their professional profile, the demographic questionnaire measured categorical profession types (i.e., specific employment settings such as public ministries, private healthcare facilities, and NGOs) rather than hierarchical professional status. Given their active roles in these diverse social service settings, the vast majority of the respondents inherently have direct or indirect professional contact with vulnerable groups, including individuals with disabilities.

### 2.2. Measures

The researchers used four different tools to collect information about the participants in their study. These tools were the “Personal Information Form”, the “Scale of Social Workers’ Attitudes Towards Disability”, the “Nathanson and Giffords Social Work Ethics Scale” and the “Empathy Level Determination Scale”.

The “Personal Information Form” was created by the researchers to gather demographic data about the participants, such as their gender, age, occupation, monthly income level, and family type. This was done in conjunction with the other measurement tools.

The “Scale of Social Workers’ Attitudes Towards Disability” was developed by Cheatham et al. (2015) and was adapted for use in Turkey by Kırlıoğlu and Kırlıoğlu (2021). The scale consists of 20 items and uses a 7-point Likert-type rating system ranging from 1 to 7 points, with one indicating “strongly disagree” and seven indicating “strongly agree”. The scale assesses four sub-dimensions: professional attitude, personal competence, equality, and professional satisfaction. Ten items of the scale are reverse scored. The professional attitude sub-dimension consists of eight items, the personal competence sub-dimension consists of six items, the equality sub-dimension consists of three items, and the professional satisfaction sub-dimension consists of three items. The scale’s reliability coefficient was calculated to be 0.87.

In a study conducted by Artan et al. (2019), Nathanson and Giffords Ethics Scale (NGES) was adapted into Turkish. NGES is a tool developed in 2013 for evaluating social work ethics. The study calculated a Cronbach α internal consistency coefficient of 0.731 for NGES, which demonstrated good validity and reliability. The study also evaluated the item-total score correlations of the 18-item scale, revealing that the correlations of the items varied between 0.119 and 0.642. The correlations between each item and the total score were found to be significant except for items 2 and 4. The study excluded these items from the analysis, resulting in an increased Cronbach’s α value of 0.760. Additionally, the factor analysis conducted for the NGES showed that the eigenvalues of the first and second factors were significantly different. Therefore, it was decided to collect all items under one factor as in the original scale.

Kaya and Çolakoğlu (2015) conducted a validity and reliability study of the Empathy Level Determination Scale, which has three dimensions: social skills, emotional response, and cognitive empathy. The scale is evaluated on a 5-point Likert scale ranging from strongly disagree (1) to strongly agree (5). The reliability coefficients for the whole scale and the three sub-dimensions were obtained as (0.78, 0.64, 0.71, 0.74) in the first stage and as (0.86, 0.61, 0.75, 0.74) in the second stage, demonstrating good reliability.

To provide clearer insight into the constructs being measured and to improve transparency for readers without direct access to the original instruments, a selection of representative sample items from each scale and their respective sub-dimensions is presented in Table 1.

### 2.3. Ethical Considerations

To conduct this study, the researchers obtained Ethics Committee permission from the Kırıkkale University Non-Interventional Ethics Committee on 21 December 2022 with the decision number 2022.12.16. They also obtained permission to use the scales from the authors via e-mail.

### 2.4. Data Analysis

During the analysis of the data, we performed the Kolmogorov–Smirnov test to check if the distribution was normal. The results indicated that the data had a normal distribution. To test the reliability of the scales, we used Cronbach’s Alpha reliability method. The reliability coefficients of the scales were 0.76 for the Social Workers’ Attitudes Towards Disability Scale, 0.60 for the Nathanson and Giffords Social Work Ethics Scale, and 0.80 for the Empathy Scale. Although the alpha value for the ethics scale (α = 0.60) obtained in the present sample is below the conventional 0.70 threshold and lower than the value reported in the original Turkish validation, values between 0.60 and 0.70 are generally considered acceptable in social science research, particularly for constructs assessing broad psychological or ethical dimensions (Hair et al., 2010; Taber, 2018).

We conducted Pearson Product Moment Correlation Coefficient analysis to determine the relationship between the research variables. For the mediation analysis, a composite total score of the Attitudes Towards Disability Scale was utilized as the dependent variable. Although the correlation matrix indicates that certain sub-dimensions (such as equality and professional satisfaction) exhibit weak or near-zero correlations with other components in this sample, the total score was constructed by aggregating all items (after appropriately reverse-scoring items 2, 5, 6, 7, 9, 10, 11, 12, 19, and 20). This aggregation aligns with the original scale developers’ and the Turkish adapters’ guidelines (Cheatham et al., 2015; Kırlıoğlu & Kırlıoğlu, 2021), who conceptualized the instrument as yielding a valid global index of professional attitudes. Utilizing the total score is theoretically justified here, as the study’s central hypotheses focus on the overarching, general attitudinal disposition of social workers toward disability rather than isolated facets, thereby providing a parsimonious dependent variable for the mediation model. We also created a regression model and tested mediation hypotheses using PROCESS v.3.3. plug-in developed by Andrew Hayes as SPSS version 25.0 macro program. The analysis was not explained only on the “*p*” significance value, and the confidence interval was calculated using the bootstrap method, which does not require a normal distribution condition. When these confidence intervals (BootLLCI and BootULCI) do not contain zero, the indirect effect is considered statistically significant.

**Figure 1:**
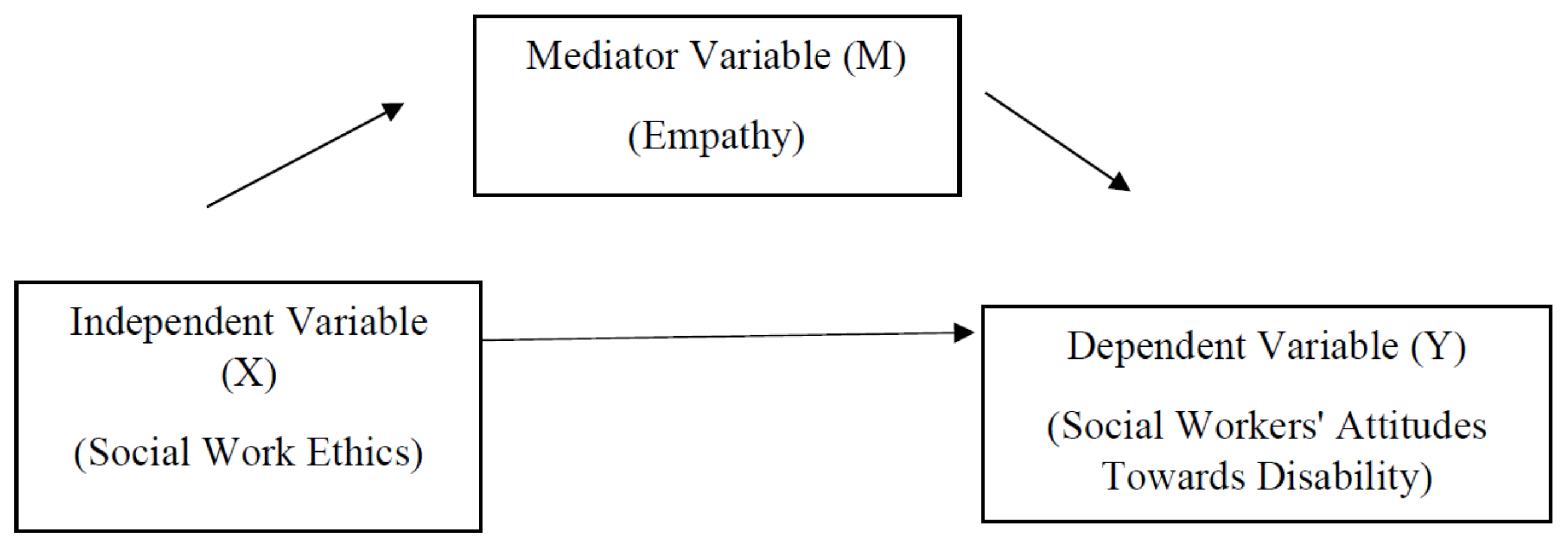
Research Model Showing the Mediation Effect.

Based on the results, we established Model 4, which was developed by Hayes (2013). In this model, social work ethics was presented as the independent variable, social workers’ attitudes towards disability as the dependent variable, and empathy as the mediating variable (see Figure 1). We used 5000 resampling with the bootstrapping method at a 95% confidence interval to evaluate the significance of indirect effects. The effects between variables were coded as a, b, and c paths and as the independent variable (X), dependent variable (Y), and mediator variable (M). Additionally, preliminary analyses were conducted to evaluate the potential impact of demographic and professional variables (such as gender, age, personal connection to disability, and profession type). As these variables did not show significant theoretical or statistical confounding effects that would alter the primary pathways, they were not included as covariates in the final PROCESS model to maintain model parsimony. Throughout the results, tables, and figures, the label “social work ethics” is utilized to refer specifically to the empirical score derived from the NGES, which operationalizes the overarching construct of professional ethical sensitivity.

## 3. Results

Correlation Analysis Results Between Attitudes Towards Disability, Nathanson and Giffords Social Work Ethics and Empathy Variables of Social Workers Participating in the Study.

When Table 2 was examined, it was determined that there was a significant positive correlation between the total score obtained from the scale of social workers’ attitudes towards disability and professional attitude, personal competence, equality, professional satisfaction, social work ethics, and empathy levels (r = 0.728; r = 0.734; r = 0.597; r = 0.545; r = 0.149; r = 0.290; *p* < 0.001).

It was determined that there was a significant positive relationship between the professional attitude sub-dimension of social workers’ attitudes towards disability and personal competence, professional satisfaction, social work ethics scale, and empathy levels (r = 0.440; r = 0.658; r = 0.285; r = 0.435; *p* < 0.001).

It was found that there was a significant positive relationship between the personal competence sub-dimension of social workers’ attitudes towards disability and equality, professional satisfaction, and empathy levels (r = 0.224; r = 0.243; r = 0.183; *p* < 0.001).

It was determined that there was a significant positive relationship between the professional satisfaction sub-dimension of social workers’ attitudes towards disability and social work ethics scale (r = 0.235; *p* < 0.001) and empathy level (r = 0.372; *p* < 0.001).

It was determined that there was a significant positive relationship between the social work ethics scale and empathy level (r = 0.342; *p* < 0.001).

Regression Analysis Results Examining the Mediating Effect of Empathy in the Relationship Between Nathanson and Giffords Social Work Ethics and Social Workers’ Attitudes Towards Disability.

When the values in Table 3 are examined, as a result of the regression analysis conducted to determine whether empathy has a mediating effect on the relationship between social work ethics and social workers’ attitudes towards disability, it is seen that the regression model for path “a” is significant (R^2^ = 0.12; *F*(1,353) = 46.66; *p* < 0.05).

**Figure 2:**
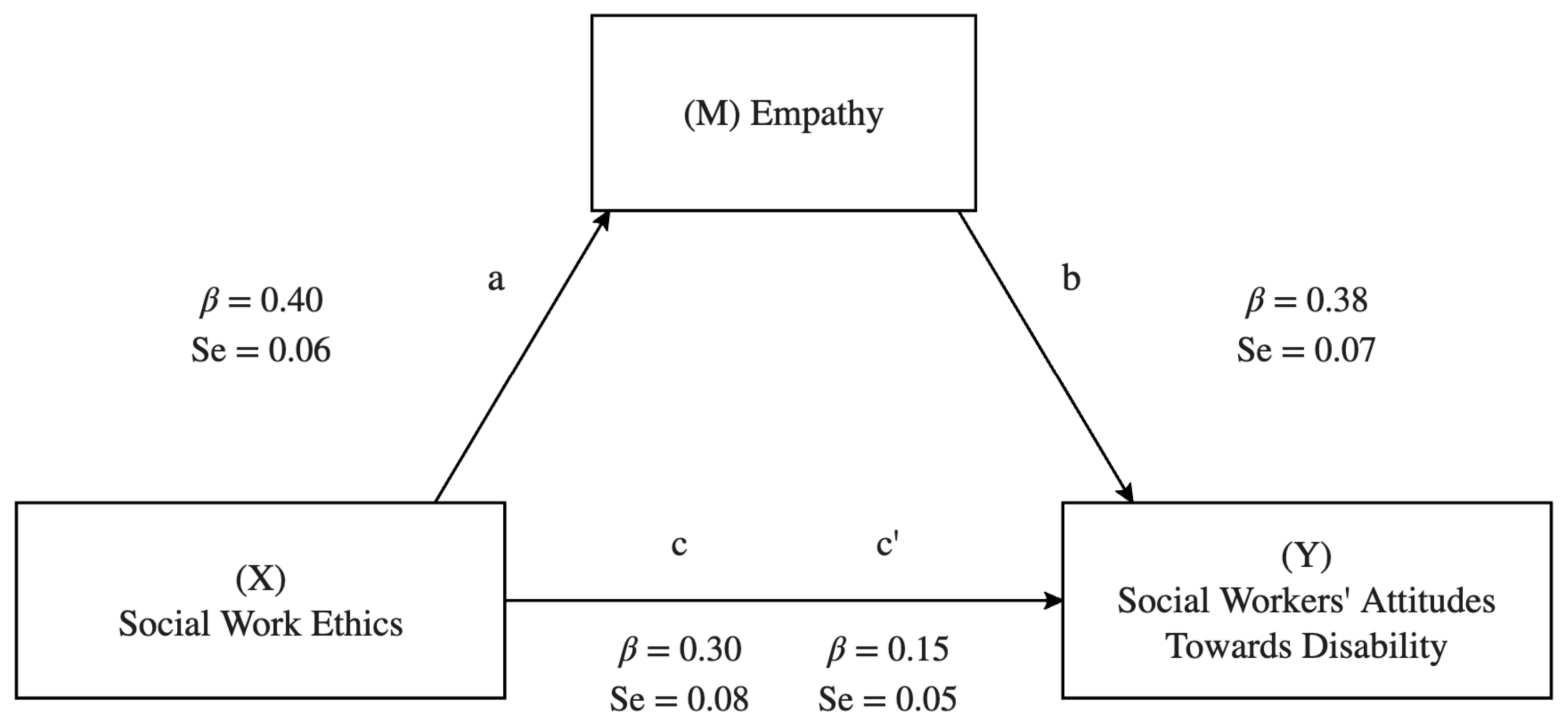
Research Model Result of the Mediation Relationship of Empathy.

When the analysis results shown in Figure 2 are examined, it was determined that social work ethics was positively associated with empathy (a = β = 0.40). When the effect of social work ethics was examined, empathy had a significant effect on social workers’ attitudes towards disability (b = β = 0.38).

In further analyses, the mediating variable of empathy was included in the model. It was observed that the initial effect of social work ethics on social workers’ attitudes towards disability changed. Specifically, the direct effect of social work ethics on attitudes towards disability (c’) was determined as 0.15. This result indicates that empathy mediates the relationship between social work ethics and social workers’ attitudes towards disability. The significance of the mediating variable was assessed through bootstrapping analysis, which revealed that the indirect effect of social work ethics on social workers’ attitudes towards disability through empathy was significant (β = 0.15, BootLLCI = 0.069, BootULCI = 0.261).

Another key finding of the study is that professional ethical sensitivity, together with empathy, accounts for 9% of the variance in the level of social workers’ attitudes towards disability (R^2^ = 0.09 *F*(2,352) = 16.77, *p* = 0.000). Based on the comprehensive analyses, the decisions regarding the study’s hypotheses are mapped as follows:

- H1 was supported, as professional ethical sensitivity demonstrated a significant positive correlation with more inclusive attitudes toward disability (r = 0.149, *p* < 0.001).
- H2 was supported, as empathy demonstrated a significant positive correlation with more inclusive attitudes toward disability (r = 0.290, *p* < 0.001).
- H3 was supported, as the PROCESS macro analysis confirmed that empathy significantly mediates the relationship between professional ethical sensitivity and attitudes toward disability (indirect effect β = 0.15, BootLLCI = 0.069, BootULCI = 0.261).

## 4. Discussion and Conclusions

The primary aim of this study was to investigate the structural relationship between professional social work ethics, empathy, and attitudes toward disability, specifically focusing on whether empathy acts as a mediating variable. The findings confirmed a strong positive correlation among all three variables. More crucially, the results of the PROCESS macro analysis revealed that while social work ethics significantly predicts positive attitudes toward disability, this relationship is partially and significantly mediated by empathy.

This core finding substantiates the “emotional bridge” hypothesis proposed in this study. Consistent with Tuncay and Sunay (2009), who emphasized that empathy is foundational across all phases of the social work intervention process, our results suggest that abstract ethical codes can be more effectively translated into genuine, inclusive professional attitudes when accompanied by emotional engagement. While cognitive knowledge of ethics provides the formal rules of conduct (the “knowing”), empathy provides the psychological connective tissue that helps integrate these rules into internalized, positive attitudes toward disabled individuals (the “feeling”). This aligns with previous assertions that employees in helping professions requiring intense face-to-face communication must develop observable empathic skills to establish vulnerable and productive client relationships (Mlcak & Zaskodna, 2008; Pedersen, 2009; Reynolds & Scott, 1999). Numerous studies across various fields—such as pediatric care and criminal justice—reiterate that empathy is central to client satisfaction and successful outcomes (Straussner & Phillips, 2005; Strug et al., 2003).

Furthermore, our findings demonstrate that individuals with high empathic capacities tend to be highly attuned to social values (de Kemp et al., 2007). In the context of disability, this attunement is vital. As highlighted by Garibay (2019) and Goulden (2025), positive practitioner attitudes are a core determinant of practice capability, directly affecting cultural safety, trust, and equitable care for disabled clients. By establishing that empathy mediates the ethics–attitude pathway, this study provides empirical evidence that fostering empathic abilities is not merely a soft skill but a critical professional competency required to prevent diagnostic harms and service disparities.

An important nuance in our results is the effect size. The mediation model indicates that social work ethics and empathy together account for 9% of the variance (R^2^ = 0.09) in attitudes toward disability. While this indirect effect is statistically significant, the relatively low total variance explained is highly informative. It indicates that professional attitudes toward disability are multifaceted and cannot be entirely captured by individual-level traits such as ethics and empathy alone. This finding suggests that systemic and organizational factors such as institutional culture, heavy caseloads, resource constraints, and broader societal ableism likely play a substantial role in shaping a social worker’s ultimate attitude in practice. Therefore, while individual empathy is necessary, it operates within a broader contextual ecosystem that also requires systemic intervention.

### 4.1. Implications for Social Work Education and Practice

The results have profound implications for social work training and policy. The partial mediation effect demonstrates that relying solely on the cognitive transmission of ethical codes and disability rights legislation in higher education is insufficient. Curricula must transcend the traditional, didactic teaching of ethics by integrating applied, affective empathy training. To bridge the gap between theoretical ethics and practiced attitudes, social work programs should heavily utilize experiential and active learning materials. Techniques such as structured role-playing, reflective supervision, and the critical analysis of media and films depicting disability experiences can simulate real-world emotional engagement. In fact, research demonstrates that students and professionals in health and social care benefit significantly from targeted empathy training (Holm, 2002; Rasoal et al., 2009). Strengthening the “empathic muscle” of social workers through such pedagogical tools can act as a robust protective mechanism against implicit bias, professional burnout, and the dehumanization of vulnerable populations.

### 4.2. Limitations and Future Research

It is important to note several limitations of this study. First, due to the cross-sectional relational survey design, the current findings represent statistical associations rather than definitive causal relationships. Second, the reliance on self-reported measures may introduce social desirability bias, particularly concerning sensitive topics like professional ethics and attitudes toward disability. Third, the study utilized a convenience sampling method, and the sample was predominantly female (67.6%). Consequently, the findings should be interpreted as associations specific to this particular sample rather than universally generalizable results representing the wider social work population. Fourth, the internal consistency of the professional ethical sensitivity measure (NGES) in the present sample was relatively low (α = 0.60). Because this construct served as the independent variable in the mediation model, this measurement attenuation likely depressed the estimated direct and indirect effects. Consequently, the modest explanatory power of the overall model (9% of variance explained) should be interpreted not only as a reflection of complex real-world organizational dynamics but also partially as a methodological artifact of this measurement attenuation.

Future research should employ longitudinal designs to clarify temporal relationships and causality between ethics education, empathy development, and attitude formation. Additionally, mixed-methods studies across diverse cultural and organizational contexts could improve generalizability. Investigating the macro-level organizational variables (e.g., agency policy, peer culture) that account for the remaining variance in professional attitudes will provide a more comprehensive understanding of how to foster disability-affirming practices in social work.

## Figures and Tables

**Table 1:** Representative Sample Items from the Measurement Instruments.

Scale and Sub-Dimensions	Sample Items
Scale of Social Workers’ Attitudes Towards Disability	
Professional attitude	“I advocate for the social inclusion of my clients with disabilities.” (Item 14)
Personal competence	“I feel uncomfortable when communicating with clients with disabilities.” (Item 10, reverse scored)
Equality:	“Individuals with disabilities are completely different from individuals without disabilities.” (Item 6, reverse scored)
Professional satisfaction	“I feel good about the effort I make to try to understand the needs of my clients with disabilities.” (Item 8)
Nathanson and Giffords Social Work Ethics Scale	“Unless required by law, a social worker should not share client information with anyone in the agency other than professionals directly involved with or vital to the client.” (Item 8)
	“A social worker should support a healthcare policy that allows access for all citizens, even if it means some people lose individual control over their healthcare options.” (Item 9)
Empathy Level Determination Scale	“Other people tell me that I am good at understanding their feelings and what they are thinking.” (Item 5)
	“I can quickly tune in to how someone else is feeling.” (Item 9)

**Table 2:** Pearson product-moment correlation coefficient results for the correlation of variables with each other.

Variables	1	2	3	4	5	6	7
Social Workers’ Attitudes Towards Disability (1)	1						
Professional attitude (2)	0.728 **	1					
Personal competence (3)	0.734 **	0.440 **	1				
Equality (4)	0.597 **	0.056	0.224 **	1			
Professional satisfaction (5)	0.545 **	0.658 **	0.243 **	−0.096	1		
NG Social work ethics scale (6)	0.149 **	0.285 **	0.090	−0.097	0.235 **	1	
Empathy level (7)	0.290 **	0.435 **	0.183 **	−0.066	0.372 **	0.342 **	1

** *p* < 0.01.

**Table 3:** Mediation analysis results regarding the mediating role of empathy in the relationship between social work ethics and social workers’ attitudes towards disability.

		Empathy (M)				Social Workers’ Attitudes Towards Disability (Y)		
Predictor Variables		β	S_e_	*p*		β	S_e_	*p*
Social work ethics (X)	a	0.40	0.06	0.000	c’	0.15	0.05	0.003
Empathy (M)		-	-	-	b	0.38	0.07	0.000
Constant	i^1^	29.12	3.37	0.000	i^2^	99.68	5.37	0.000
R^2^ = 0.12, *F*(1,353) = 46.66, *p* = 0.000						R^2^ = 0.09, *F*(2,352) = 16.77, *p* = 0.000		

Note: i^1^ and i^2^ represent the constant (intercept) values for the respective regression models.

## Data Availability

The data presented in this study are available on request from the corresponding author. The data are not publicly available due to privacy and ethical restrictions.
